# Physiology and Chemistry

**Published:** 1859-03

**Authors:** 


CHIEFLY FRENCH, GERMAN, AND ITALIAN; OR, BI-MONTHLY NOVELTIES IN MEDICAL, SURGICAL, OBSTETRICAL, AND CHEMICAL SCIENCE. BY EMIL FISCHER, M.D.

### PHYSIOLOGY AND CHEMISTRY.

### I.—*On the Suspension of the Radial Pulse in Forced Extension of the Arm.* By DR. A. VERNEUIL.

THE suspension of the radial pulse is always observed when the extension of the forearm on the arm is actively or passively exaggerated. The difficulty experienced in exploring the ulnar artery does not allow an explicit statement in regard to the suspension of its pulsation. The anatomical dispositions harmonize with direct experience, so as to prove that in forced extension the arteries of the forearm and hand receive but little blood. In this position the aponeurotic expansion of the biceps and the brachialis anticus press the humeral artery against the convex projection formed by the coronoid process of the ulna; the artery is thus flattened for a length of about two centimetres, and, according to the experiments of M. Verneuil, this phenomenon must take place in a great number of physiological movements, particularly in mechanics—a fact which may serve to explain the predisposition shown by the termination of the brachial artery to spontaneous arteriectasis. This fact could also be made use of as a means of arresting the flow of blood in cases of arterial hemorrhage from wounds of the forearm, when there is no assistant present to compress the humeral artery.—(*Journal de Physiologie*, tome i. p. 506, and *Archives Générales*, November, 1858.)

### II.—*Some Experimental Researches on the Effects of Cold on the Human Body.* By MM. THOLOZAN and BROWN-SÉQUARD.

A series of researches permits the authors to draw up the following propositions:—1. If our extremities are exposed to the action of cold water, they can in a very short time lose a considerable portion of their temperature, (according to our experiments, from ten to eighteen degrees.) 2. After an extremity has lost much of its temperature, (from ten to eighteen degrees,) it does not regain it before the end of forty-five minutes or an hour in an atmosphere varying from twelve to eighteen degrees. 3. Contrary to the opinion of Edwards, the lowering of the temperature of a small part of the human body has no sensible influence upon the general temperature. 4. The lowering of the temperature of one hand can produce considerable falling of the temperature of the other, without the general temperature of the body being sensibly diminished.

Dr. Brown-Séquard has ascertained that the latter phenomenon becomes more marked if the immersed hand is the seat of more intense pain, and if the temperature of the air in which the other hand is kept is less elevated; it is also proportional to the contraction of the vessels of the hand not immersed, and it is this contraction exclusively which produces the falling of the temperature. This phenomenon is an example of reflex action upon the blood-vessels, their muscles contracting under the influence of an irritation applied to the sensitive nerves of another extremity; it is further remarkable, that this reflex action takes place only between homologous parts, and that the immersion of the hand, for instance, into cold water, exercises no appreciable influence upon the temperature of the feet.—(*Journal de la Physiologie*, tome i. p. 497, and *Archives Générales*, November, 1858.)

### III.—*On the Formation of Vivianite in the Animal Organism.* By M. J. NICKLES.

M. Schiff has proved, by chemical analysis, that the blue color which pus sometimes presents is owing to phosphate of iron in an amorphous state. It is this same salt which gives the blue color to animal remains which have been interred for a long time. The demonstration of this fact is owing to M. Nickles, who has found in human bones phosphate of iron crystallized in the form peculiar to the vivianite of mineralogists.—(*Journal de Pharmacie et de Chimie*, June, 1858.)

